# A porous 3D-RGO@MWCNT hybrid material as Li–S battery cathode

**DOI:** 10.3762/bjnano.10.52

**Published:** 2019-02-21

**Authors:** Yongguang Zhang, Jun Ren, Yan Zhao, Taizhe Tan, Fuxing Yin, Yichao Wang

**Affiliations:** 1 School of Materials Science and Engineering, Hebei University of Technology, Tianjin 300130, China; 2 Synergy Innovation Institute of GDUT, Heyuan, Guangdong Province, China; 3 School of Life and Environmental Sciences, Deakin University, Geelong, Vic 3216, Australia

**Keywords:** carbon nanotubes, energy storage and conversion, Li–S batteries, nanocomposites

## Abstract

In this work, a unique three-dimensional (3D) structured carbon-based composite was synthesized. In the composite, multiwalled carbon nanotubes (MWCNT) form a lattice matrix in which porous spherical reduced graphene oxide (RGO) completes the 3D structure. When used in Li–S batteries, the 3D porous lattice matrix not only accommodates a high content of sulfur, but also induces a confinement effect towards polysulfide, and thereby reduces the “shuttle effect”. The as-prepared S-3D-RGO@MWCNT composite delivers an initial specific capacity of 1102 mAh·g^−1^. After 200 charging/discharge cycles, a capacity of 805 mAh·g^−1^ and a coulombic efficiency of 98% were maintained, implying the shuttle effect was greatly suppressed by the composite matrix. In addition, the S-3D-RGO@MWCNT composite also exhibits an excellent rate capability.

## Introduction

Li–S batteries are notable for their high theoretical specific capacity (1675 mAh·g^−1^) and energy density (2600 Wh·kg^−1^). Sulfur is an abundant element, enabling Li–S batteries to be highly competitive among the various battery technologies. The actual application of Li–S batteries, however, is hindered by several challenges, i.e., i) the poor conductivity of sulfur and ii) the “shuttle effect” of polysulfides (Li_2_S*_x_*, 4 < *x* ≤ 8) [1–4]. To achieve a high specific capacity, a sulfur cathode with high electrical conductivity and high sulfur loading is necessary. The shuttle effect will result in rapid fading of the capacity and coulombic efficiency during the cycling process. Therefore, the development of a sulfur cathode that can “withhold” sulfur and reduce the shuttle effect, together with a high conductivity and sulfur loading is essential for the practical implementation of Li–S batteries [5–7].

To overcome the above-mentioned challenges in Li–S batteries, many strategies have been proposed [8–12]. For example, metal oxides, such as TiO_2_, ZnO, MnO_2_, and SiO_2_, were reported to provide active sites for strong S–metal bonding that have been reported to suppress the shuttle effect in polysulfides [13–16]. Moreover, designing metal oxides into various unique morphologies, e.g., hollow structures, can also provide a physical (or structural) confinement for sulfur [17]. Metal-oxide materials, however, have a major drawback, i.e., their electronic conductivity is very low [16,18]. To improve the conductivity of the sulfur cathode, it was typically composited with carbon materials [19–23]. Moreover, the high surface area of the carbon substrate was beneficial for a higher sulfur loading [24–25]. Since sulfur is the major active ingredient in the Li–S cathode, adding more non-sulfur components, such as metal oxides, in the cathode will result in a lower specific capacity.

Therefore, the present study will focus on the development of a pure carbon material for the Li–S cathode. It was believed that a carbon-based material network with specific morphology will not only allow for a high sulfur loading but will also provide both the chemical and physical restraints on the polysulfide shuttle effect. In the previous report, we synthesized porous 3D reduced graphene oxide (3D-RGO), showing a reversible capacity of 790 mAh·g^−1^ (at 0.2*C*) after 200 cycles [26]. It has been reported that three-dimensional carbon nanotubes/graphene–sulfur (3DCGS) is an excellent cathode template, revealing a final capacity of 975 mAh·g^−1^ after 200 cycles [24]. Carbon nanotubes (CNTs) can be used to adjust structure and density of the pores of the composite while improving the electrical conductivity. Following such a strategy, we developed a unique three-dimensional structured carbon-based composite material, referred to as 3D-RGO@MWCNT. Multiwalled carbon nanotubes (MWCNTs) form a lattice network for the composite that is supported by porous spherical reduced graphene oxide (RGO). Furthermore, the functional groups on RGO provide bonding sites for the active sulfur material. The 3D porous carbon structure enabled high sulfur loading and confined the sulfur within the 3D MWCNT network and the porous spherical RGO. Moreover, such a 3D structure can buffer the volume expansion/shrinkage of the sulfur cathode during charge and discharge cycles. Lastly, the electrochemical performance of the resulting S-3D-RGO@MWCNT cathode was evaluated in Li–S batteries.

## Results and Discussion

**Figure 1:**
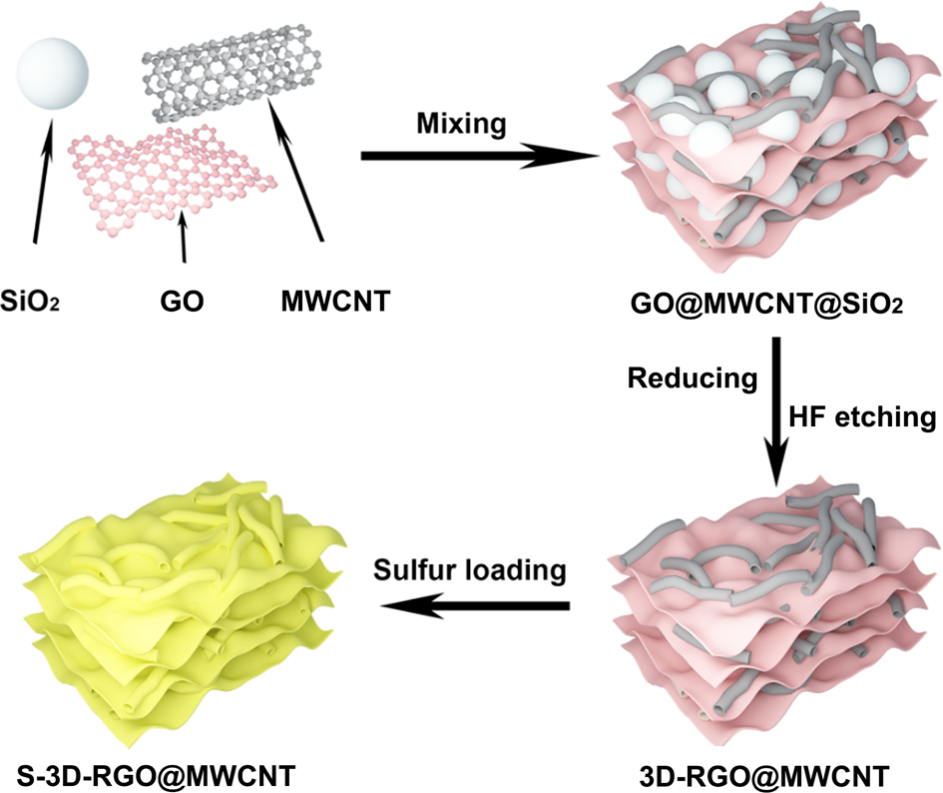
Synthesis of S-3D-RGO@MWCNT.

**Figure 2:**
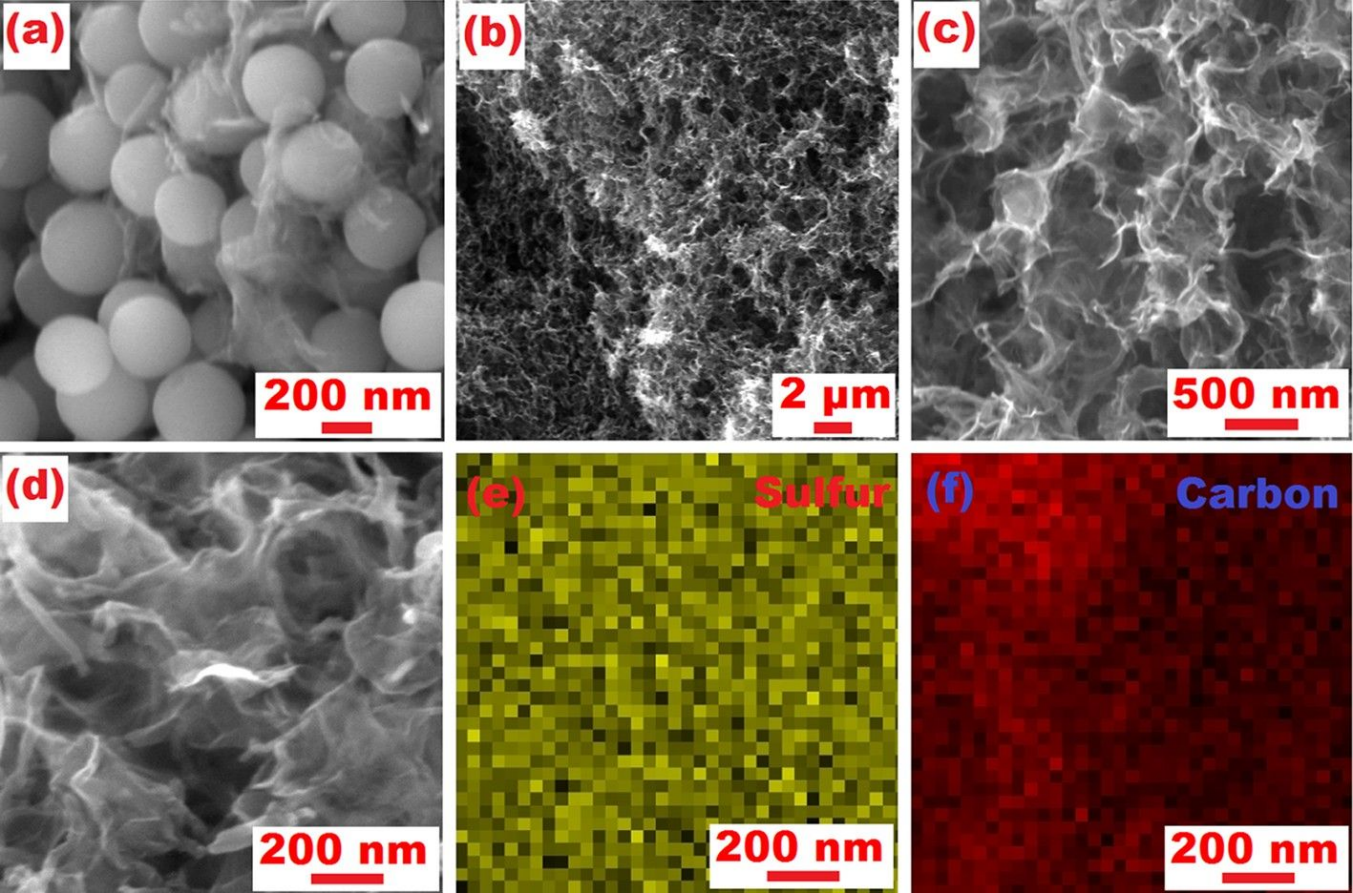
SEM images of (a) RGO@MWCNT@SiO_2_, (b, c) 3D-RGO@MWCNT at different magnifications and (d) S-3D-RGO@MWCNT, and corresponding elemental maps of (e) sulfur and (f) carbon.

**Figure 3:**
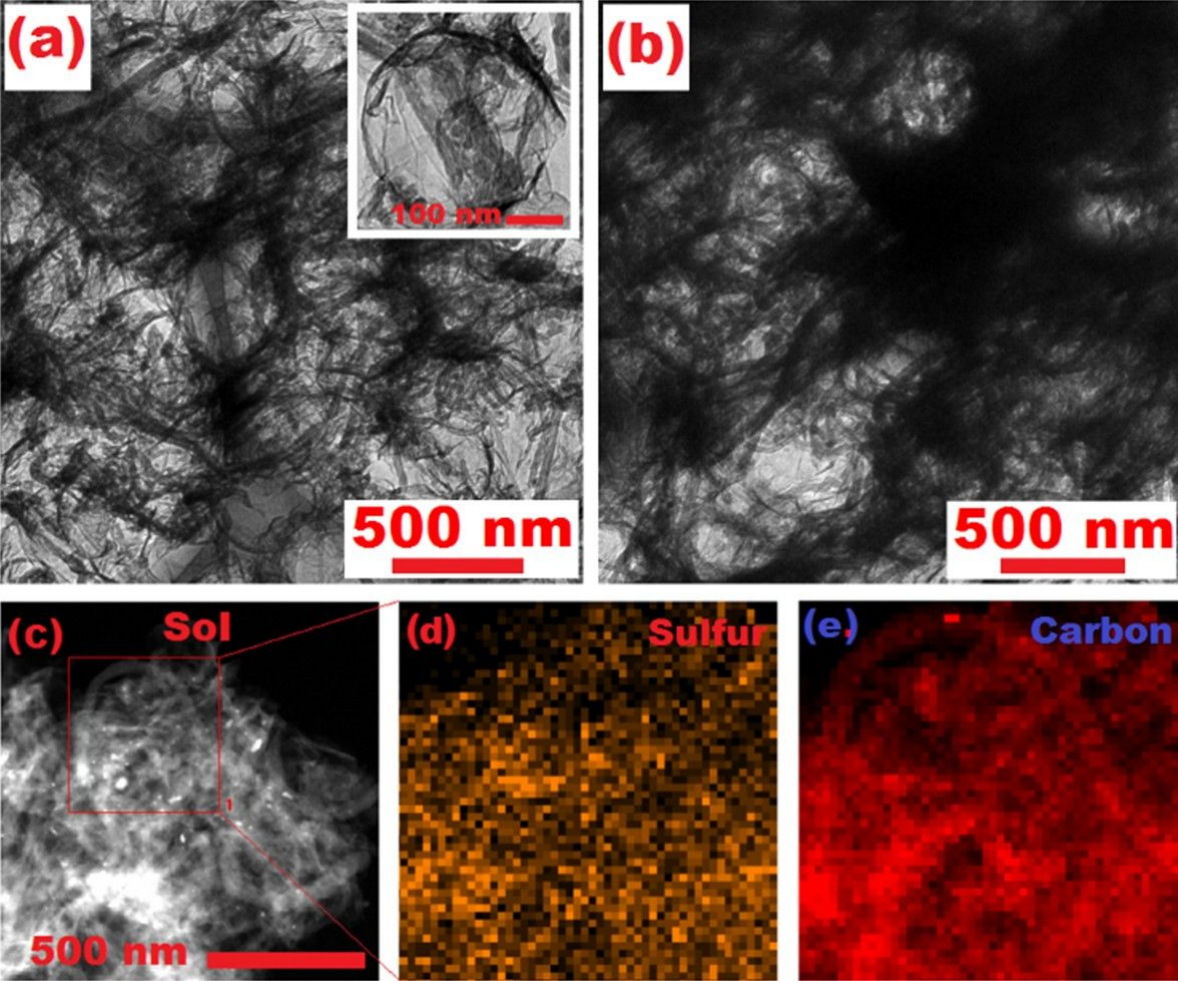
TEM images of (a) 3D-RGO@MWCNT with two different magnifications, (b) S-3D-RGO@MWCNT, (c–e) TEM mapping of (d) sulfur and (e) carbon corresponding to the area outlined by the red square in the TEM image of (c).

The synthesis of the 3D-RGO@MWCNT composite is illustrated in Figure 1, highlighting the 3D porous RGO structure and the MWCNT lattice matrix. The SEM images confirmed that the precursor composite, RGO@MWCNT@SiO_2_, contained 200–300 nm SiO_2_ particles that were successfully encased by RGO and MWCNTs (Figure 2a). After HF etching, a 3D-RGO@MWCNT was obtained (Figure 2b,c). The porous spherical indents (ca. 200 nm) remained after the removal of SiO_2_ (Figure 3a). Furthermore, after sulfur loading, both SEM (Figure 2d) and TEM (Figure 3b) images revealed that the structure remained in the resulting S-3D-RGO@MWCNT composite. The EDS elemental mapping validated the successful and uniform loading of sulfur into the composite (Figure 2e and Figure 3d). The 3D structure provided: i) higher usable surface area for a higher sulfur loading, ii) empty spaces between the pores and the lattice matrix to reduce the shuttle effect by acting as a lithium polysulfide reservoir, and iii) additional empty spaces to buffer the volume expansion/shrinkage in the charge and discharge processes enhancing the cycling performance of the battery. The electrochemical performance of the S-3D-RGO@MWCNT composite will be discussed later in the electrochemical analysis.

**Figure 4:**
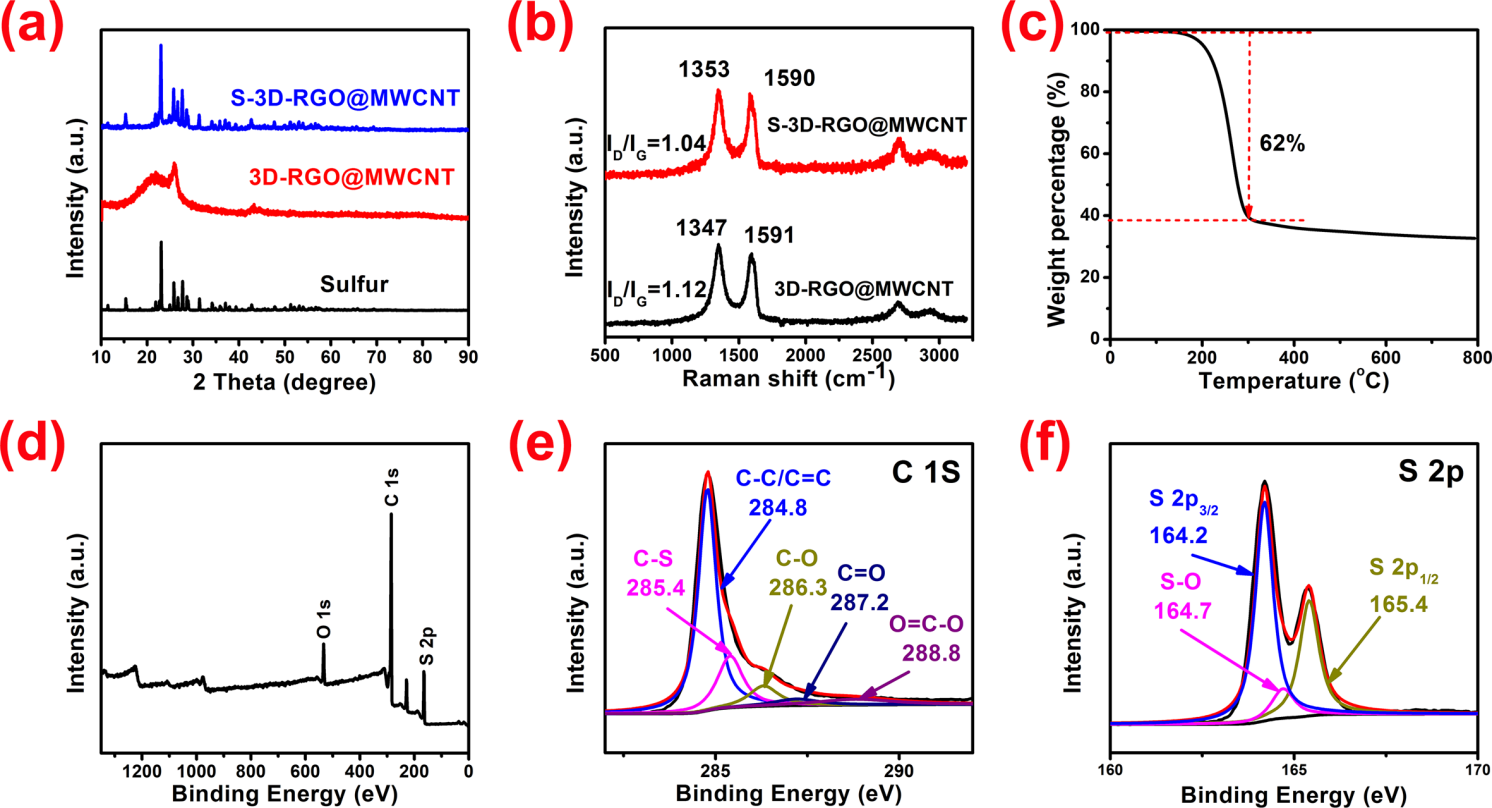
(a) XRD patterns of sulfur, 3D-RGO@MWCNT and S-3D-RGO@MWCNT; (b) Raman spectra of 3D-RGO@MWCNT and S-3D-RGO@MWCNT; (c) TGA of S-3D-RGO@MWCNT; (d) The XPS survey spectrum of S-3D-RGO@MWCNT composite; high-resolution XPS spectra of (e) C 1s, (f) S 2p.

Figure 4a presents the XRD patterns for pure S, 3D-RGO@MWCNT and the S-3D-RGO@MWCNT composite. The XRD pattern of 3D-RGO@MWCNT exhibits two broad characteristic peaks of RGO at around 22° and 43°. Moreover, a diffraction peak around 26° for 3D-RGO@MWCNT corresponds to the MWCNTs. In the XRD pattern of S-3D-RGO@MWCNT, the major characteristic peaks of crystalline sulfur are observed, which further confirm the preservation of crystalline sulfur in the composite after adding sulfur. The Raman spectra demonstrates that the ratio *I*_D_/*I*_G_ decreased from 1.12 in 3D-RGO@MWCNT to 1.04 in S-3D-RGO@MWCNT (Figure 4b), implying that the defects in 3D-RGO@MWCNT were filled or occupied by sulfur [3]. This is also supported by the C 1s XPS pattern of 3D-RGO@MWCNT, in which a C–S bonding state (285.4 eV) is observed (Figure 4d). The O–C=O (288.8 eV), C=O (287.2 eV) and C–O (286.3 eV) peaks in the C 1s pattern confirm the oxide nature of RGO sheets. In addition to the C–S bonding, O-containing groups also help retain sulfur via S–O bonding, as revealed by the peak located at 164.7 eV in the S 2p spectrum (Figure 4e). The strong chemical bonding of C–S and S–O can immobilize sulfur and polysulfides within S-3D-RGO@MWCNT, reducing the shuttle effect and improving the cycling life of Li–S batteries. The thermogravimetric analysis (TGA) analysis (Figure 4f) shows that the S-3D-RGO@MWCNT composite exhibits a very high weight loss (62 wt %) between 30 and 300 °C, confirming that a great amount of sulfur can be stored in the structure.

**Figure 5:**
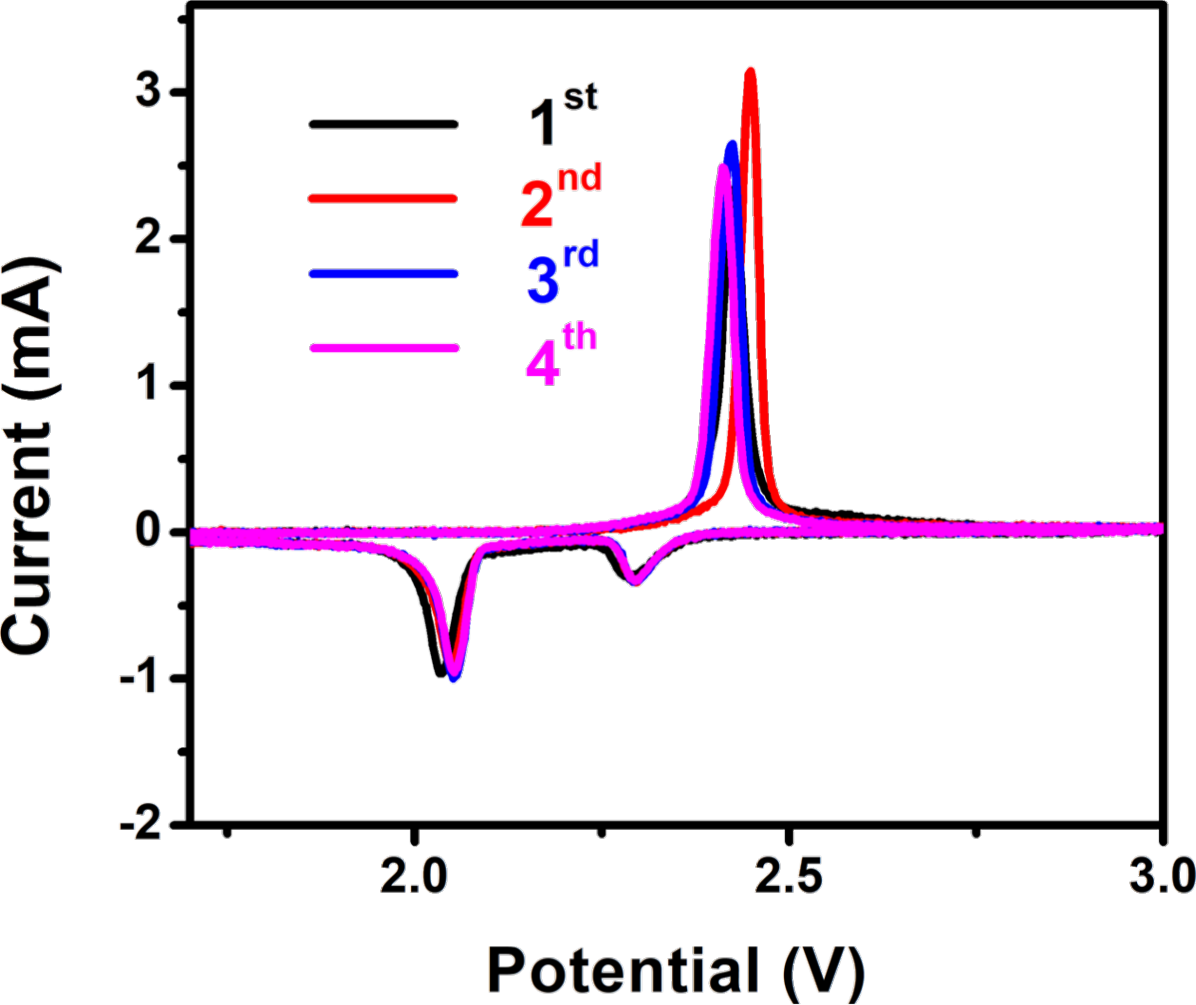
CV curves of the S-3D-RGO@MWCNT cathode at 0.1 mV·s^−1^ in the first four cycles.

Figure 5 displays the first four CV cycles of S-3D-RGO@MWCNT cathode at 0.1 mV·s^−1^. During the cathodic cycle, the peaks around 2.30 and 2.05 V correspond to the transformation of elemental sulfur to long-chain polysulfides (Li_2_S*_n_*, *n* ≥ 4) and the reduction to short-chain polysulfides (*n* < 4), respectively. On the anodic side, the peak located at around 2.40 V corresponds to the oxidation of lithium polysulfides (Li_2_S*_n_*, *n* < 4) and Li_2_S to Li_2_S_8_. It can be seen that during the cycling, the anodic peak shifts to a lower voltage, whereas the cathodic peaks remain almost unchanged. These results suggest the superior discharge stability of the S-3D-RGO@MWCNT cathode.

**Figure 6:**
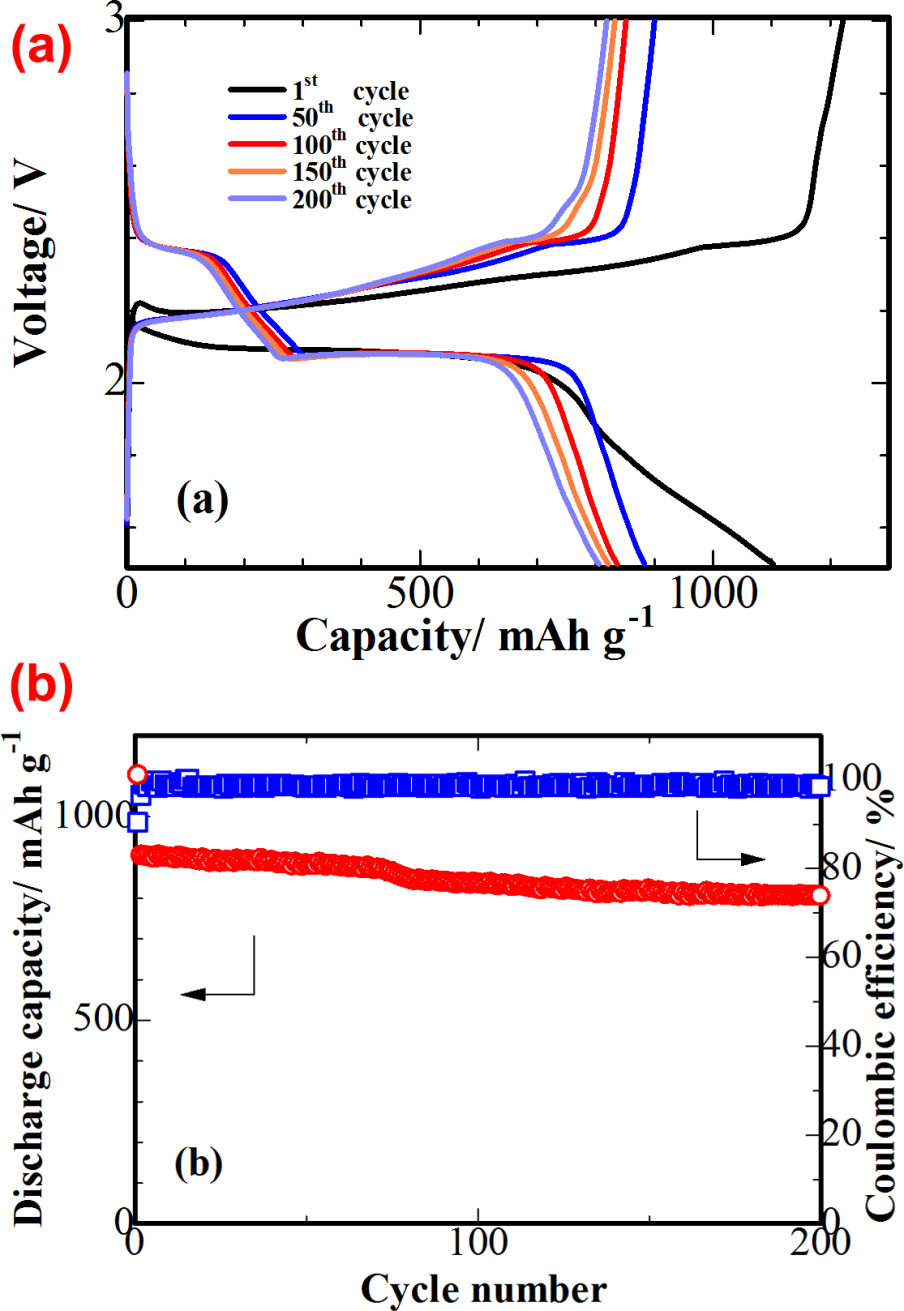
(a) CV measurement of the S-3D-RGO@MWCNT cathode (1st, 50th, 100th, 150th and 200th cycle) at 1*C*; (b) cycling performance of the S-3D-RGO@MWCNT cathode at 1*C* for 200 cycles.

Figure 6a shows the charge and discharge voltage profiles of the S-3D-RGO@MWCNT cathode measured at 1*C*. The plateaus on the discharge (2.30 and 2.05 V) and charge (2.40 V) profiles are consistent with those observed in the CV cycles. The voltage plateaus were preserved after 200 cycles, confirming the excellent electrochemical stability of sulfur in the 3D structure of S-3D-RGO@MWCNT. The S-3D-RGO@MWCNT cathode exhibits an initial specific discharge capacity of 1102 mAh·g^−1^ and a retained reversible capacity of 805 mAh·g^−1^ after 200 cycles. This result concurs with that observed in the cycling performance of the S-3D-RGO@MWCNT cathode (Figure 6b). The discharge/charge coulombic efficiency was maintained at approximately 98% after 200 cycles. The cycling performance of S-3D-RGO@MWCNT indicates the efficient confinement of sulfur preventing the loss of active material through the shuttle effect.

**Figure 7:**
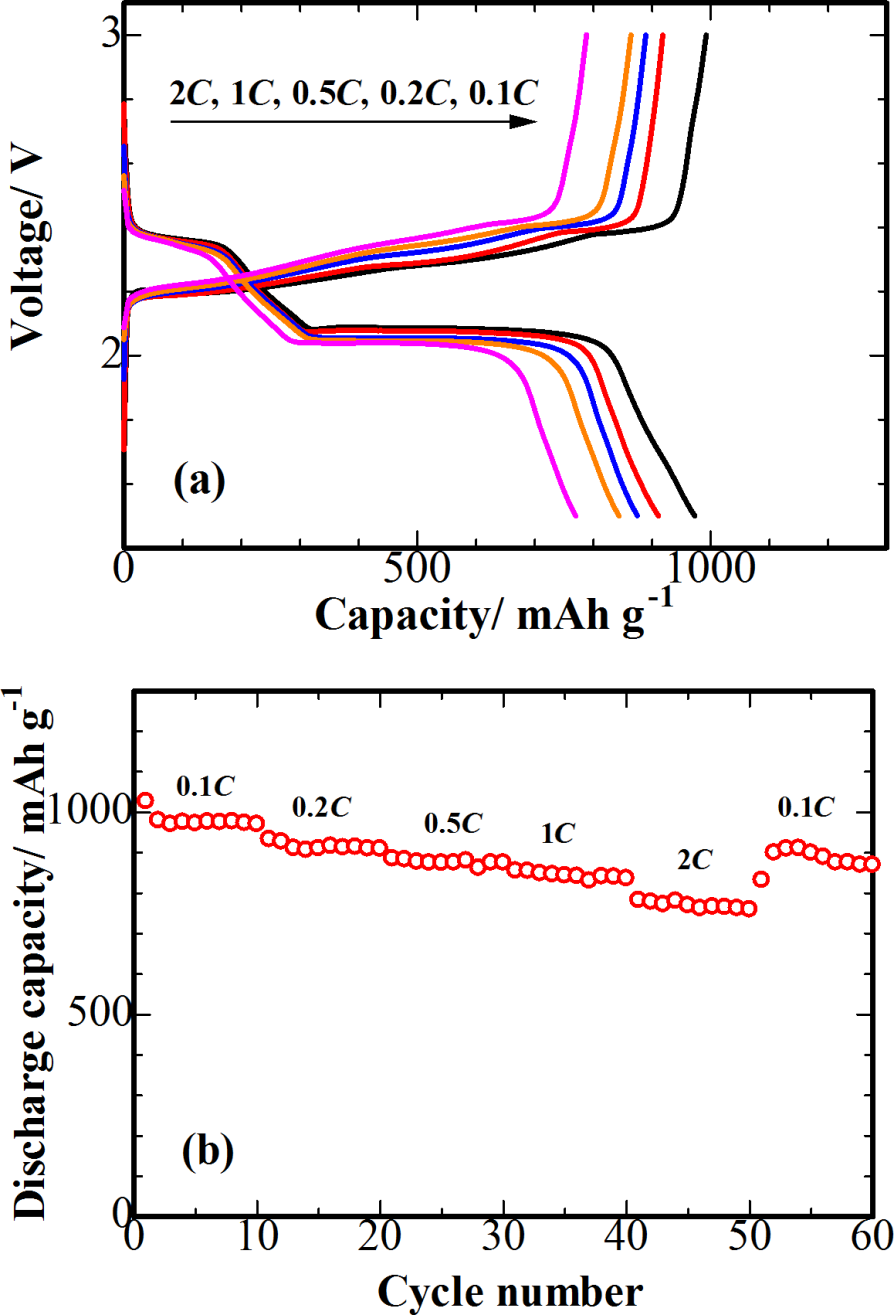
(a, b) Specific capacity and rate performance of S-3D-RGO@MWCNT cathode at different C-rate, ranging from 0.1*C* to 2*C*.

Figure 7a reveals the charge–discharge voltage profiles of the batteries measured at various rates across the voltage range of 1.5 to 3.0 V. A two-plateau behaviour of the discharge profiles was observed at all current densities, which is consistent with the CV curves peaks (Figure 5). As the current increases from 0.1*C* to 2*C*, the polarization of the plateaus becomes higher, implying a slow decrease in the kinetic efficiency of the reaction process. This may have resulted from a weak influence of the current density on lower discharge plateau [27]. The rate capability of the S-3D-RGO@MWCNT cathode is examined in greater detail in Figure 7b. The impressive rate capability of the S-3D-RGO@MWCNT cathode was verified. Although a decrease of discharge capacity was observed when the current rate increases, a capacity of 770 mAh·g^−1^ was still obtained at 2*C*. When the current returned back to 0.1*C*, a capacity of 889 mAh·g^−1^ was preserved. These observations reveal that the 3D structure upheld the excellent rate performance of the S-3D-RGO@MWCNT cathode.

**Figure 8:**
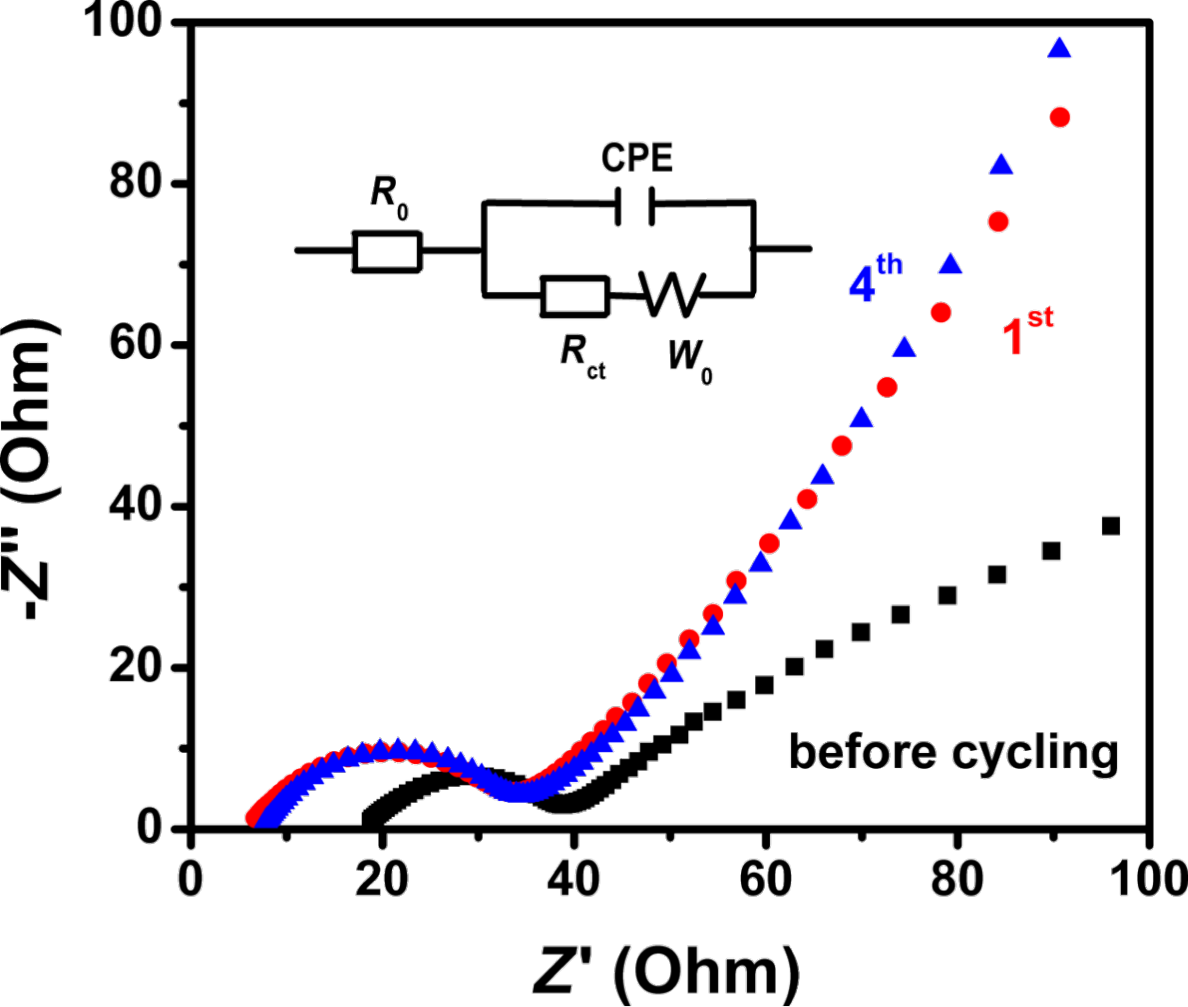
Nyquist plots of S-3D-RGO@MWCNT cathode and the equivalent circuit model (inset).

The changes in the conductivity during cycling a Li–S battery equipped with the S-3D-RGO@MWCNT cathode, were investigated using electrochemical impedance spectroscopy (EIS). Figure 8 presents the Nyquist plots for the Li–S cell assessed before cycling, and after the 1st and the 4th cycle. In the high-frequency region the *x*-intercept is attributed to the contact resistance (*R*_0_), and the semicircle is attributed to the charge-transfer resistance (*R*_ct_) at the electrode/electrolyte interface. Finally, the inclined slope in the low-frequency region is associated with the Warburg impedance (W) [28], which correlates to the Li^+^ transportation process. Notably, there is a significant shift in the impedance curves before and after cycling. The primary reason for the decrease in the contact resistance after the initial cycle may be the redispersion of sulfur. The significant shift in the Warburg element indicates an improved Li^+^ diffusivity [29]. *R*_ct_ increases slightly, then stabilizes after the initial cycle, which agrees with the cyclability data. The fitted values of *R*_0_ and *R*_ct_ for the S-3D-RGO@MWCNT cathode are tabulated in Table 1. The impedance curves of the 1st and the 4th cycle are similar and become very stable, indicating the enhanced electrochemical performance of the S-3D-RGO@MWCNT cathode, which can be attributed to its 3D porous lattice matrix structure and the facilitation of rapid Li^+^ diffusion.

**Table 1:** Impedance parameters of the S-3D-RGO@MWCNT cathode.

cycle number	*R*_0_ (Ω)	*R*_ct_ (Ω)

before cycling	18.08	22.11
1st cycle	6.32	26.4
4th cycle	7.62	26.49

## Conclusion

In summary, a unique S-3D-RGO@MWCNT composite, consisting of porous spherical RGO integrated within a MWCNT lattice matrix, was successfully synthesized. The as-prepared S-3D-RGO@MWCNT cathode exhibited a very good electrochemical performance and cycle stability. This can be attributed to i) the conductive network inherently found in the RGO sheets and MWCNTs, which ensured efficient charge transfer within the cathode, ii) the 3D porous spherical RGO possessing a high surface area and pore volume to accommodate a high sulfur content; and iii) the interconnected pores in the spherical RGO and the lattice matrix formed by MWCNTs, which act as polysulfide reservoirs to alleviate the shuttle effect, and thereby improving the cycling stability of the battery. Lastly, the interconnected pores ensured the rapid Li^+^ diffusion during the discharge/charge process, and therefore were beneficial for reducing the internal resistance and improving the electrochemical properties.

## Experimental

### Synthesis of 3D-RGO@MWCNT composite

The synthesis of 3D-RGO@MWCNT composite consists of the following steps: i) the preparation of monodispersed SiO_2_ spherical particles using Stober’s method [30]; ii) the preparation of graphene oxide (GO) using Hummers method [31]; iii) the incorporation of MWCNTs; iv) the reduction of GO, and v) SiO_2_ etching by HF. Firstly, monodispersed SiO_2_ spheres with diameters of 200–300 nm were prepared. After washing and drying, the SiO_2_ sphere particles was subsequently dispersed in DI water at a concentration of 50 mg·mL^−1^ (suspension A). Secondly, the GO from Hummer’s method was dispersed into DI water at a concentration of 2 mg·mL^−1^, and subsequently mixed with a 2 mg·mL^−1^ MWCNT suspension at a mass ratio of 1:1. The as-prepared GO@MWCNT suspension was afterwards mixed with suspension A and volumetric ratio of 3:1 resulting in GO@MWCNT@SiO_2_ (suspension B). After sonicated for 30 min, sodium erythorbate was added to suspension B and heated in an oil bath for 2 h. The sodium erythorbate was removed by washing with DI water, while SiO_2_ was etched away by subsequent soaking in 10% HF for a week. Lastly, HF was also rinsed out with DI water and ethanol. After drying the compound at 60 °C for 12 h, the 3D-RGO@MWCNT composite was obtained.

### Synthesis of S-3D-RGO@MWCNT composite and S-cathode

The as-prepared 3D-RGO@MWCNT was mixed with nano-sulfur at a mass ratio of 1:2. The resulting sample was heated at 155 °C for 12 h in a nitrogen-filled autoclave producing the S-3D-RGO@MWCNT composite. The cathode was fabricated by coating a slurry of S-3D-RGO@MWCNT, polyvinylidene fluoride (PVDF) and carbon black (mass ratio 8:1:1) on a carbon-coated Al foil.

### Materials characterization

X-ray diffraction (XRD) patterns of the as-prepared 3D-RGO@MWCNT composite were obtained using XRD (SmartLab, Rigaku Corporation) with Cu Ka radiation. X-ray photoelectron spectroscopy (XPS, Shimadzy Axis Ultra) was applied to investigate the chemical valence states and compositions of the sample. Scanning electron microscopy (SEM, Hitachi S4800) and high-resolution transmission electron microscopy (HRTEM, JEOL JEM-2100F) images were used for investigating surface topology. The content of sulfur in the S-3D-RGO@MWCNT composite was confirmed using thermogravimetric analysis (TGA, SHIMADZU DTG-60) in Ar atmosphere. Raman spectra were recorded on Raman spectrometer (Raman, Renishaw) using 532 nm radiation.

### Electrochemical measurements

CR2025 coin batteries were assembled using S-3D-RGO@MWCNT as the cathode, 1 M lithium bistrifluoromethanesulfonimide and 0.1 M LiNO_3_ in a mixed solution of DME-DOL (1:1 by volume) as electrolyte, a Li foil as anode, and a Celgard 2300 membrane as separator. The cycling performances of the Li–S battery was investigated using a battery testing station (Neware, Shenzhen) in potential range of 1.5–3.0 V. The electrochemical workstation (Princeton, VersaSTAT 4) was used to evaluate cyclic voltammetry (CV) also in a potential range of 1.5–3.0 V. Electrochemical impedance spectroscopy (EIS) was carried out in the frequency range from 10^−2^ to 10^5^ Hz.

## Supporting Information

File 1 Additional experimental data.

## References

[1] He J, Chen Y, Lv W, Wen K, Li P, Wang Z, Zhang W, Qin W, He W (2016). ACS Energy Lett.

[2] Mahmood N, Hou Y (2014). Adv Sci.

[3] Zheng S, Wen Y, Zhu Y, Han Z, Wang J, Yang J, Wang C (2014). Adv Energy Mater.

[4] He J, Chen Y, Lv W, Wen K, Li P, Qi F, Wang Z, Zhang W, Li Y, Qin W (2016). J Power Sources.

[5] Barchasz C, Molton F, Duboc C, Leprêtre J-C, Patoux S, Alloin F (2012). Anal Chem (Washington, DC, U S).

[6] Mikhaylik Y V, Akridge J R (2003). J Electrochem Soc.

[7] Manthiram A, Fu Y, Chung S-H, Zu C, Su Y-S (2014). Chem Rev.

[8] Guo Z, Nie H, Yang Z, Hua W, Ruan C, Chan D, Ge M, Chen X, Huang S (2018). Adv Sci.

[9] Rehman S, Gu X, Khan K, Mahmood N, Yang W, Huang X, Guo S, Hou Y (2016). Adv Energy Mater.

[10] He J, Lv W, Chen Y, Xiong J, Wen K, Xu C, Zhang W, Li Y, Qin W, He W (2018). J Mater Chem A.

[11] Mahmood N, Zhang C, Yin H, Hou Y (2014). J Mater Chem A.

[12] He J, Chen Y, Manthiram A (2018). Energy Environ Sci.

[13] Huang J-Q, Wang Z, Xu Z-L, Chong W G, Qin X, Wang X, Kim J-K (2016). ACS Appl Mater Interfaces.

[14] Gu X, Tong C-j, Wen B, Liu L-m, Lai C, Zhang S (2016). Electrochim Acta.

[15] Wang S, Yang Z, Zhang H, Tan H, Yu J, Wu J (2013). Electrochim Acta.

[16] Qu Q, Gao T, Zheng H, Wang Y, Li X, Li X, Chen J, Han Y, Shao J, Zheng H (2015). Adv Mater Interfaces.

[17] Gu X, Wang Y, Lai C, Qiu J, Li S, Hou Y, Martens W, Mahmood N, Zhang S (2015). Nano Res.

[18] Li Z, Zhang J, Lou X W D (2015). Angew Chem, Int Ed.

[19] Gnana kumar G, Chung S-H, Raj kumar T, Manthiram A (2018). ACS Appl Mater Interfaces.

[20] He J, Chen Y, Lv W, Wen K, Xu C, Zhang W, Li Y, Qin W, He W (2016). ACS Nano.

[21] He J, Chen Y, Manthiram A (2017). Adv Mater (Weinheim, Ger).

[22] He J, Chen Y, Manthiram A (2018). iScience.

[23] Zhou W, Wang C, Zhang Q, Abruña H D, He Y, Wang J, Mao S X, Xiao X (2015). Adv Energy Mater.

[24] He J, Chen Y, Li P, Fu F, Wang Z, Zhang W (2015). J Mater Chem A.

[25] He J, Chen Y, Lv W, Wen K, Xu C, Zhang W, Qin W, He W (2016). ACS Energy Lett.

[26] Zhang Y, Sun L, Li H, Tan T, Li J (2018). J Alloys Compd.

[27] Qian W, Gao Q, Zhang H, Tian W, Li Z, Tan Y (2017). Electrochim Acta.

[28] Martha S K, Markovsky B, Grinblat J, Gofer Y, Haik O, Zinigrad E, Aurbach D, Drezen T, Wang D, Deghenghi G (2009). J Electrochem Soc.

[29] Zhang Y, Zhao Y, Bakenov Z, Tuiyebayeva M, Konarov A, Chen P (2014). Electrochim Acta.

[30] Philipse A P (1989). J Mater Sci Lett.

[31] Wang X, Lu C, Peng H, Zhang X, Wang Z, Wang G (2016). J Power Sources.

