# Supplementary material for: A porous 3D-RGO@MWCNT hybrid material as Li–S battery cathode
Source: Beilstein J Nanotechnol. 2019 Feb 21;10:514–21. doi: 10.3762/bjnano.10.52 (PMC6404391; doi:10.3762/bjnano.10.52)
Supplement: File 1 — Additional experimental data. [file Beilstein_J_Nanotechnol-10-514-s001.pdf]

BEILSTEIN JOURNAL OF NANOTECHNOLOGY

Supporting Information

for

A porous 3D-RGO@MWCNT hybrid material as Li–S battery cathode

Yongguang Zhang, Jun Ren, Yan Zhao, Taizhe Tan, Fuxing Yin and Yichao Wang

Beilstein J. Nanotechnol. **2019,** *10,* 514–521. doi:10.3762/bjnano.10.52

Additional experimental data

License and Terms: This is a supporting information file under the terms of the Creative Commons Attribution License (http://creativecommons.org/licenses/by/4.0). Please note that the reuse, redistribution and reproduction in particular requires that the authors and source are credited.
The license is subject to the *Beilstein Journal of Nanotechnology* terms and conditions: (https://www.beilstein-journals.org/bjnano)

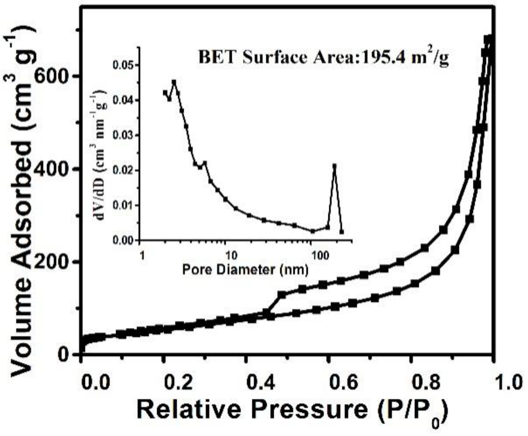

Figure S1: Nitrogen adsorption/desorption isotherms for 3D-RGO@MWCNT. Inset: Pore size distribution of the 3D-RGO@MWCNT.

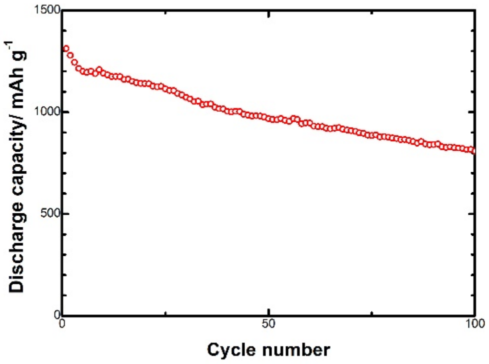

Figure S2: Cycling performance of the S-3D-RGO@MWCNT cathode with a sulfur content of 74% at 1*C* for 100 cycles.
